# Treatment expectancy and credibility as predictors of concentrated exposure treatment outcomes in patients with difficult-to-treat obsessive-compulsive disorder

**DOI:** 10.1186/s12888-025-06737-z

**Published:** 2025-03-25

**Authors:** Håvard Berg, Kristian Tjelle, Bjarne Hansen, Stian Solem, Thröstur Björgvinsson, Gerd Kvale, Kristen Hagen

**Affiliations:** 1 Department of Psychiatry, Møre and Romsdal Hospital Trust, Molde, Norway; 2 Department of Psychology, Norwegian University of Science and Technology, Trondheim, Norway; 3 Bergen Center for Brain Plasticity, Haukeland University Hospital, Bergen, Norway; 4 Department of Psychosocial Sciences, University of Bergen, Bergen, Norway; 5 Department of Psychiatry, Harvard Medical School, Boston, USA; 6 McLean Hospital, Belmont, USA; 7 Department of Clinical Psychology, University of Bergen, Bergen, Norway; 8 Department of Mental Health, Norwegian University of Science and Technology, Trondheim, Norway

**Keywords:** OCD, Treatment expectancy, Treatment credibility, cET, Difficult-to-treat, B4DT

## Abstract

**Background:**

Treatment readiness factors, such as treatment credibility and expectancy, are postulated to be predictors of outcomes within the context of cognitive behavioral therapy (CBT). Concentrated exposure therapy (cET) is a form of short-term, intensive, exposure-based CBT that has shown promising results. This study investigated whether treatment expectancy and credibility predict cET treatment outcomes in patients with difficult-to-treat (nonresponders and patients with relapse following CBT) obsessive-compulsive disorder (OCD).

**Methods:**

A total of 163 patients underwent 4 days of cET treatment. Treatment credibility and expectancy were measured using the Credibility/Expectancy Questionnaire (CEQ) prior to the start of treatment. OCD symptom severity was measured using the Yale-Brown Obsessive Compulsive Scale (Y-BOCS) at pretreatment, posttreatment, 3-month follow-up, and 1-year follow-up. Work- and social functioning were measured before treatment and at the 1-year follow-up.

**Results:**

Higher CEQ scores were significantly associated with lower Y-BOCS score at posttreatment and follow-up after controlling for age, sex, and pretreatment OCD, anxiety, and depression levels. The CEQ scores were also significantly associated with work- and social functioning at the 1-year follow-up. A receiver operating characteristic analysis suggested a mean item cutoff point of 92.5 (0-100 scale) for the CEQ, and 87% of the patients classified as having high expectancy had a positive treatment response.

**Conclusions:**

This study confirmed that treatment expectancy and credibility are predictors of cET outcomes in patients with OCD. Higher scores on the CEQ were linked to better treatment results, both immediately and up to one year later. These insights highlight the need to consider patients’ attitudes toward treatment in the early treatment phase.

**Trial registration:**

ClinicalTrials.gov identifier: NCT02656342 (First registered: 2015-11-30).

## Background

Cognitive behavioral therapy (CBT) focusing on exposure therapy with ritual prevention (ERP; [1]) is considered the treatment of choice for obsessive-compulsive disorder (OCD) [2, 3]. ERP is regarded as efficacious [4], but studies have shown that up to 30% of patients drop out of the treatment program [5], 35–40% do not respond to the treatment [6, 7], and more than 50% fail to achieve long-term recovery [8, 9]. The search for enhanced OCD treatment has led to the development of various treatment formats, ranging from internet-based treatments to intensive and concentrated group treatments [10, 11, 12, 13]. The Bergen 4-day treatment (B4DT) program is a concentrated, exposure-based CBT treatment in which patients are treated over four consecutive days [14]. The B4DT program has shown promising results in a series of trials [14, 15, 16, 17, 18, 19, 20, 21, 22, 23, 24].

Increasing knowledge about the factors associated with the response to treatment in patients with OCD is important for improving treatment [25, 26]. Treatment credibility and expectancy are concepts of treatment readiness assumed to be associated with treatment outcomes [27], but there is no research on its role in patients with a history of no response or relapse. The Credibility/Expectancy Questionnaire (CEQ; [28]) is used to measure patients’ readiness for a specific treatment. The CEQ consists of two topics: treatment credibility is defined as how logical and believable the treatment rationale is as perceived by a patient; and treatment expectancy is defined as a patient´s dedication to completing the treatment and how strongly the patient believes the treatment will be beneficial in helping them [28].

A recent systematic review investigated different elements of the therapeutic relationship as predictors of treatment outcomes in patients receiving CBT for anxiety disorders [29]. This meta-analysis identified four studies that employed the CEQ, although none of them included OCD patients. Among these studies, three revealed a positive correlation between CEQ scores and treatment outcomes, indicating a modest to moderate effect [30, 31, 32]. A study by Vogel et al. [33] with 37 patients receiving ERP revealed that expectancy that the treatment would help them with their problem (single item) was close to significant as a predictor of the Yale-Brown Obsessive Compulsive Scale (Y-BOCS) score posttreatment, but not with the 1-year follow-up, after controlling for therapeutic alliance and motivation. Conversely, in one study of 84 individuals diagnosed with various anxiety disorders, the CEQ score was not related to treatment outcomes [34].

Research on the predictive validity of treatment credibility/expectancy in OCD has shown mixed results. One study [35] found that the six item CEQ [36] score predicted posttreatment severity according to the Y-BOCS score after controlling for baseline symptoms in a study of 28 patients receiving ERP for OCD. Another study found that the six item CEQ predicted posttreatment Y-BOCS scores after controlling for baseline severity in a sample of 30 patients receiving ERP or ERP combined with motivational interviewing [37]. A third study showed that expectancy, as measured by patient expectancy ratings (“rate how much you think the behavioral treatment will be helpful in reducing your: 1. Obsessions 2. Compulsions 3. General distress”) on a scale ranging from 0 to 8, did not predict treatment outcomes in patients receiving ERP (*n* = 54) or mindfulness-based ERP (*n* = 54) [38].

Findings from studies on the association between credibility/expectancy and treatment outcome have been mixed also for studies using other patient groups. One study on the CEQ using non-OCD patients found that expectancy predicted outcome whereas credibility did not [36]. However, a meta-analysis found a significant correlation (*r* =.12) between credibility and treatment outcome [39]. Also, a recent study found that an increase in the patients’ perception of treatment credibility (3 items) may lead to an improvement of symptom severity across sessions in CBT treatment for patients with anxiety or affective disorders [40].

No studies have investigated treatment credibility and expectancy in a sample of difficult-to-treat patients or while using a concentrated CBT format. Therefore, the aim of this study was to investigate whether treatment credibility and expectancy predict treatment outcomes in a group of difficult-to-treat OCD patients receiving concentrated exposure therapy (cET). cET, with its short timeframe, entails more control over the treatment process and reduces influence from external variables. In addition, we wanted to investigate whether treatment credibility and expectancy predict work- and social functioning. The main hypothesis was that treatment credibility and expectancy would predict improvement in OCD symptoms and work- and social adjustment. Furthermore, as credibility/expectancy ratings are easily administered in the clinic, this study also tested the predictive utility of using CEQ cutoff scores in relation to treatment response and remission status.

## Methods

### Design and procedure

This study contains secondary analyses of data from a trial testing whether D-cycloserine potentiated treatment outcomes for difficult-to-treat OCD patients receiving cET [41]. The primary study used a triple-blind, three-armed, placebo-controlled design in which patients within each stratum were randomized to receive 100 mg D-cycloserine, 250 mg D-cycloserine, or placebo at a 2:2:1 ratio. Participants were informed that the main treatment was cET, and that DCS was a potential adjunct to enhance the effects of ERP. CEQ was administered specifically with reference to cET. There were no significant differences in treatment effects among the conditions at any time point (*p* values from 0.15 to 0.92). The groups’ CEQ scores were also similar (*p* =.67). Therefore, this study used the total sample for statistical analyses. The study followed the Consolidated Standards of Reporting Trials (CONSORT) guidelines. The trial protocol for the original study is available as supportive information.

### Participants

A total of 163 patients were included in the trial. Potential participants were assessed by local OCD teams as part of ordinary clinical practice. Candidates had to meet the criteria for OCD according to the Diagnostic and Statistical Manual of Mental Disorders-Fifth Edition (DSM-5) and have a Y-BOCS score of 16 points or more. Patients had to be at least 18 years old, fluent in Norwegian, and capable of receiving treatment in an outpatient clinic to be included in this study. Participants had either not responded to previous treatment or relapsed. The participants’ previous ERP treatment had to consist of at least six hours. In addition, at least four weeks had to have passed since the participants’ last treatment. A patient was defined as relapsing if they had at least a 35% increase in the Y-BOCS score and a Clinical Global Impression– Improvement (CBI-I)) score of six (“much worse”) or greater [42]. Nonresponders were classified as having less than 35% improvement and a Y-BOCS score of 16 points or more. A total of 38.7% of the patients did not respond to their last treatment for OCD, while 61.3% of the patients relapsed following their last treatment.

Patients who met any of the following criteria were excluded from the study: had ongoing substance abuse/dependence, bipolar disorder, psychosis, suicidal ideation or plans, or instability in the antidepressant dosage within the last 12 weeks; were unwilling to maintain a stable dosage during the four intervention days; were unwilling to abstain from anxiety-reducing substances (such as anxiolytics and alcohol) during the two exposure days; had intellectual disability (as indicated by previous medical history); or resided in a location more than one hour away from the treatment location by car or train. The exclusion criteria for patients who were administered D-cycloserine included pregnancy or breastfeeding, renal impairment, hypersensitivity to D-cycloserine, porphyria, and epilepsy.

The sample had a mean age of 34.6 years (10.9), and the majority of participants were female (71.8%). The average duration of OCD was 16.2 (10.2) years. In total, 113 (69.3%) participants had comorbid disorders. The most common comorbid disorders included generalized anxiety disorder (31.9%) and major depressive disorder (31.3%). Almost half of the patients (44.7%) received some type of disability benefit indicating affected work ability; 34.8% were currently employed, and 20.5% were students. Seventy-six patients (46.6%) were treated with psychotropic medication prior to the start of treatment. Most of the patients were on a treatment regimen using selective serotonin reuptake inhibitor (SSRI) medications (68.4%). Days since finishing last ERP treatment was 729.6 (*SD* = 772.9, Range = 32–4718). A summary of the sample’s characteristics is shown in Table 1.

**Table 1:** Pretreatment patient characteristics (*N* = 163)

Characteristic	M (SD)/% (*n*)
Age	34.6 (10.9)
Female sex	71.8 (117)
Duration of OCD (years)	16.2 (10.2)
Duration of last treatment (hours)	26.6 (11.0)
Any comorbid disorder	69.3 (113)
Number of comorbid disorders	1.7 (1.9)
Reported OCD in the family	39.3 (64)
Years in school	11.9 (3.9)
Employment status	
Work	34.8 (56)
Student	20.4 (33)
Disability	44.9 (74)
Use of any psychotropic medication	46.6 (76)
Use of SSRIs	31.9 (52)
CEQ score	
Total	362.78 (33.97)
Treatment logic	92.66 (11.25)
Recommend treatment	97.17 (6.17)
Dedication to treatment	87.15 (13.65)
Benefits from treatment	85.79 (13.65)
Pretreatment Y-BOCS score	27.03 (3.86)
WSAS, pretreatment	20.98 (8.44)

Note. OCD = Obsessive-Compulsive Disorder, CEQ = Credibility/ Expectancy Questionnaire, WSAS = Work and Social Adjustment Scale, Y-BOCS = Yale-Brown Obsessive Compulsive Scale, SSRI = Selective Serotonin Reuptake Inhibitor

The study was approved by the Regional Committee for Medical Research Ethics for Southeast Norway (REK Southeast: 2013/195). Written consent was obtained from all participants. The participants were informed that their participation was voluntary and that they could withdraw from the study at any point without affecting their treatment. The inclusion of patients lasted from January 2016 to August 2017.

### Treatment

The patients received the B4DT program [14], which is cET delivered across four consecutive days. The first day consisted of providing psychoeducation in a group setting with 3–6 patients and planning relevant exposure tasks. The following two days consisted of individually tailored and therapist-assisted exposure and response prevention. In brief, this means that patients actively seek out situations that elicit a fear response and then prevent their immediate and automatic fear-reducing actions from occurring. By doing this, patients learn new ways to cope with fear-eliciting situations. Exposure tasks were adapted specifically for each patient, and they were dynamic in the sense that they could be changed in cooperation with the therapist during the treatment to further increase the efficacy of each exposure task.

Adherence measurements investigating each patient’s level of anxiety, their degree of ritual prevention and the general usefulness of the current exposure tasks were utilized by both the patient and the therapist to ensure that the patient followed the treatment principals and that the exposure tasks were relevant to the individual treatment goals. Patient rated adherence [26] showed a significant correlation with CEQ scores, *r* =.35, *p* <.001. The patients continued the exposure tasks after leaving the clinic at the end of Days two and three and received phone support from the therapist at prearranged points during the afternoon. The patients came together as a group intermittently during the days to share their experiences and to support each other.

The last day was about patients linking the core theoretical elements of the treatment with their own experiences and planning relevant self-administered exposure tasks for the following three weeks. A more detailed description of the treatment procedure is given by Launes et al. [20].

### Measures

*The study used a modified version of the CEQ* [28] which consisted of four items measuring how credible and logical a patient believes a treatment to be, as well as the patient´s expectation of improvement after the treatment. The modification involved using a 0-100 scale for all items, rephrasing item 3, and removing item 5, and one of the two items about expected benefits from treatment. The four items were as follows: (1) How logical does this treatment seem to you? (2) Would you recommend the treatment to a friend? (3) What is the likelihood that you will fully commit to the treatment and follow the treatment recommendations? and (4) What do you think the likelihood is that you will benefit from the treatment? The four CEQ item scores were summed into a total CEQ score. The internal consistency was good (α = 0.73).

Different versions of the CEQ have been used with number of items ranging from 1 to 6, and rating scales using 1–9 scales and 0-100 ratings. The original CEQ had five items and the CEQ scores were found to be lower following rationales presented for control conditions than in therapy conditions [28]. Later a 6-item version was published finding that expectancy was associated with outcome whereas credibility was not [36].

The CEQ was administered to participants during a semi-structured interview as an integral component of the pretreatment assessment protocol. Prior to the administration of the CEQ, a concise introduction to the B4DT treatment, including an explanation of its underlying rationale, was provided to the patients. Participants were briefed on the treatment’s structural framework and were informed that it primarily consisted of exposure and response prevention techniques. The patients were made aware that the treatment would entail deliberate exposure to anxiety-inducing stimuli without engaging in response-prevention behaviors throughout the treatment program. An independent assessor administered the CEQ immediately after the introductory briefing on B4DT. Participants were explicitly informed that their responses on the CEQ were specific to their expectations of the B4DT. Therapists were blinded to the CEQ responses to maintain treatment integrity. We ensured participants understood that their feedback was collected solely for research purposes and would not influence their treatment process.

*The Y-BOCS* [43] is a semistructured clinical interview measuring OCD severity. It consists of 10 items scored on a scale ranging from 0 to 4, with 5 items each for obsessions and compulsions. The Y-BOCS is widely recognized as the gold standard for assessing OCD severity. The psychometric properties of the scale are well established, with good internal consistency [43, 44]. Cronbach’s alpha was 0.73.

*The Work and Social Adjustment Scale* (WSAS; [45]) is a questionnaire containing five items. The items focus on an individual’s impairment in areas of work, social and private activities and functioning at home and in close relationships. The items are individually rated on a 9-point scale ranging from 0 (not at all) to 8 (very severe). Total scores range from 0 to 40 points, with higher scores indicating greater functional impairment. The WSAS has good internal consistency and test–retest reliability [46]. In this study, measurements were taken before treatment and at the 1-year follow-up. Cronbach’s alpha was 0.86.

The Patient Health Questionnaire-9 (PHQ-9; [47]) is a self-administered questionnaire for assessing depressive symptoms. It consists of nine items, with each item rated on a scale from 0 to 3, culminating in a total score from 0 to 27 points. A higher score indicates increased symptom severity. Across diverse settings, the PHQ-9 has consistently exhibited strong psychometric properties [48, 49]. Cronbach’s alpha was 0.86.

The Generalized Anxiety Disorder 7-item (GAD-7; [50]) scale is a self-report questionnaire for assessing the severity of generalized anxiety symptoms. The scale comprises seven items rated on a scale from 0 to 3, yielding a total score ranging from 0 to 21 points. Higher scores indicate increased symptom severity. The GAD-7 has shown good psychometric properties in diverse settings [48, 51]. Cronbach’s alpha was 0.82.

### Statistical analyses

A repeated-measures ANOVA was conducted to primarily investigate the interaction effect between time and CEQ score (covariate) on Y-BOCS and WSAS scores. For the Y-BOCS analysis four times of assessment were included (pretreatment, posttreatment, 3-month follow-up, and 12-month follow-up). For the WSAS analysis, only pre-treatment and 12-month follow-up data was available. When the sphericity assumption was violated, corrections were applied using the Greenhouse‒Geisser correction.

Additionally, hierarchical regression analyses were conducted to investigate the association between the CEQ score and OCD symptoms at posttreatment, 3-month follow-up, and 1-year follow-up. The regression analyses controlled for pretreatment levels of the outcome measure, sex, age, and pretreatment levels of anxiety and depression. The regression was repeated using the WSAS score at the 1-year follow-up as the dependent variable. Finally, we conducted a receiver operating characteristic (ROC) analysis to test the predictive validity of using CEQ cutoff scores in relation to treatment response and remission status at post-treatment.

To address missing data and include all participants in our analyses, we employed the expectation maximization (EM) method in SPSS version 29, which is a suitable approach when less than 25% of the data are missing and the absence of data is random. Overall, 7.8% of the participants had missing data. A Little’s Missing Completely at Random (MCAR) test was performed, indicating that the data were missing at random (χ^2^ = 479.22 (df = 431), *p* =.054.

).

## Results

A repeated-measures ANOVA was conducted to evaluate the interaction effect of the CEQ score on the Y-BOCS and WSAS scores across time. The analysis revealed a significant interaction effect of the CEQ score on the Y-BOCS score (*F*(2.71, 435.46) = 9.827, *p* <.001, *η*_p_² = 0.16). For the WSAS score, there was also a significant interaction effect (*F*(1, 161) = 9.369, *p* =.003, *η*_p_² = 0.06). The results showed that higher CEQ scores were associated with improvements in OCD symptoms and work and social functioning. Correlations between Y-BOCS and WSAS suggested that the two instruments were associated at pre-treatment (*r* =.29, *p* <.001) and at 12-month follow-up (*r* =.77, *p* <.001).

According to our regression analysis, the CEQ score was a significant predictor across all assessments (Table 2). At posttreatment, the CEQ score was negatively associated with the Y-BOCS score, explaining 12% of the variance. At the 3-month follow-up, a similar pattern was observed, with a negative association between the CEQ score and Y-BOCS score, again accounting for 12% of the variance. At the 12-month follow-up, the strength of the association was reduced, but the CEQ score remained a significant predictor of the Y-BOCS score, explaining 3% of the variance. Furthermore, at the 12-month follow-up, the CEQ score was negatively associated with the WSAS score, accounting for 2% of the variance.

**Table 2:** Regression analysis including the Y-BOCS and WSAS scores as outcome measures

Predictor	β	t	*p*	sr
**Posttreatment Y-BOCS score**				
Y-BOCS score, pretreatment	0.10	1.37	0.174	0.10
Age	0.02	0.29	0.769	0.02
Male sex	0.00	-0.02	0.984	− 0.00
GAD-7 score, pretreatment	− 0.01	-0.13	0.896	− 0.01
PHQ-9 score, pretreatment	0.13	1.32	0.189	0.10
CEQ score	− 0.35	-4.69	< 0.001	− 0.35
**3-month follow-up Y-BOCS score**				
Y-BOCS score, pretreatment	0.22	2.97	0.003	0.21
Age	0.04	0.53	0.596	0.04
Male sex	0.03	0.47	0.642	0.03
GAD-7 score, pretreatment	0.06	0.69	0.490	0.05
PHQ-9 score, pretreatment	0.08	0.88	0.378	0.06
CEQ score	− 0.34	-4.74	< 0.001	− 0.34
**12-month follow-up Y-BOCS score**				
Y-BOCS score, pretreatment	0.09	1.16	0.249	0.09
Age	− 0.03	-0.34	0.735	− 0.03
Male sex	0.03	0.41	0.679	0.03
GAD-7 score, pretreatment	0.17	1.77	0.079	0.13
PHQ-9 score, pretreatment	0.10	1.01	0.313	0.08
CEQ score	− 0.17	-2.21	0.029	− 0.17
**12-month follow-up WSAS score**				
WSAS score, pretreatment	0.49	6.10	< 0.001	0.41
Age	− 0.03	-0.37	0.710	− 0.03
Male sex	0.02	0.22	0.827	0.01
GAD-7 score, pretreatment	0.18	2.01	0.046	0.14
PHQ-9 score, pretreatment	− 0.10	-1.12	0.264	− 0.08
CEQ score	− 0.15	-2.12	0.036	− 0.14

Note. Y-BOCS = Yale-Brown Obsessive Compulsive Scale; CEQ = Credibility/Expectancy Questionnaire; WSAS = Warwick-Edinburgh Mental Wellbeing Scale; GAD-7 = Generalized Anxiety Disorder-7; PHQ-9 = Patient Health Questionnaire-9

**Fig. 1:**
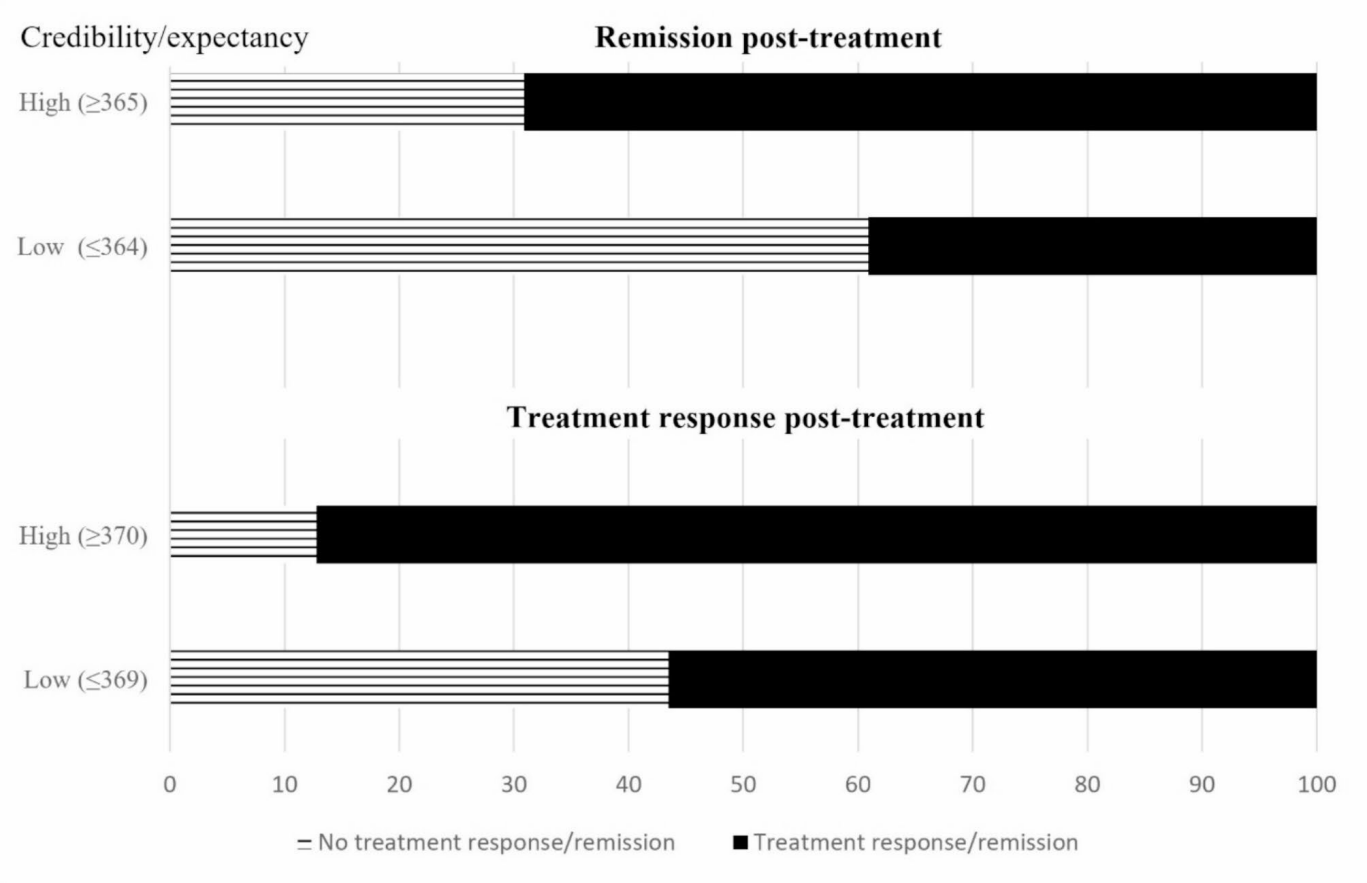
Treatment outcomes for patients with high or low treatment credibility/expectancy

The ROC analysis suggested a cutoff point of 369 for the CEQ. This entailed a mean item score of 92.5. The median scores of the four items were 100 for items 1 (logical) and 2 (recommend), and 90 for items 3 (dedication) and 4 (benefit). The sensitivity was 0.678 and specificity of 0.286 when using a Y-BOCS posttreatment score lower than 16 points as the grouping variable indicating treatment response (area under the curve = 0.74). The CEQ grouping variable was significantly associated with treatment response (χ^2^_(1)_ = 19.62, *p* <.001). Most patients classified as having high treatment expectancy had a positive treatment response (87.2%), while the corresponding rate for patients with low treatment expectancy was 56.5%. A summary of the groups’ treatment response rates is illustrated in Fig. 1.

The ROC analysis was repeated using remission (Y-BOCS scores of 12 or less) as the grouping variable. The ROC analysis suggested a cutoff point of 365 (area under the curve = 0.68). The sensitivity was 0.707 while specificity was 0.408. The CEQ grouping variable was significantly associated with remission at post-treatment (χ^2^_(1)_ = 14.59, *p* <.001). Patients with low CEQ scores had 39.1% remission, while patients with high scores had 69.1% remission.

## Discussion

The patients reported that the B4DT program seemed very credible and that they had a strong dedication to the treatment and a high treatment expectancy (mean scores of approximately 90). The regression analysis revealed that the CEQ score was a significant predictor of the Y-BOCS score at posttreatment, at 3-month follow-up, and at 1-year follow-up, even after controlling for the pretreatment Y-BOCS score, depression, anxiety, sex, and age. This is the first study to assess the impact of treatment expectancy and credibility using a concentrated or intensive format and with a difficult-to-treat sample. Although the follow-up period in the present study was longer than that in most other studies, the results are in line with previous studies using the CEQ [30, 31, 32, 33], although the same pattern was not found in LeBeau et al. [34].

The regression analysis revealed that the CEQ score was also a significant predictor of work and social functioning at the 1-year follow-up. The amount of explained variance was small, so there is a need for additional studies to establish whether this is a robust finding and whether the finding can be replicated for different treatment formats and different patient populations. It is worth noting the high levels of treatment credibility and treatment expectancy, despite the patients’ previous lack of success with OCD treatment. This finding suggested a generally positive attitude toward the potential benefits of the treatment that was not negatively affected by previous exposure-based CBT attempts. The positive attitudes to treatment could be related to the treatment rationale, previous treatment successes (for relapsed patients), and ideas about what could be done differently (learning from past failures) during the upcoming treatment.

Several studies indicated that assessing expectancy may increase patients’ motivational language early on, leading to an enhanced treatment process with better outcomes [52, 53, 54]. This, in turn, can contribute to an enhanced therapeutic experience and ultimately result in better treatment outcomes. The findings from these studies highlight the potential value of assessing and addressing patients’ treatment expectations and perceptions early in the therapeutic process. The ROC analysis suggested that patients who achieved a positive treatment response reported very high treatment credibility and had high expectancy before the treatment was started. The suggested cutoff point of 92.5 indicates that patients should preferably receive a score of 90 to 100 points on the individual CEQ items. Therapists should therefore address any doubts and ambivalence when patients have CEQ scores lower than 90 points.

We consider it important to address previous treatment attempts before initiating another ERP treatment to try and understand why the patient did not respond or what caused the relapse. Developing a treatment rationale incorporating this information could be important and raise credibility and expectations. Previous research has shown that ERP in general is associated with higher CEQ scores than stress-management training [55]. If patients report low CEQ scores (e.g. mean scores below 90), the therapist should address these doubts and problem solve them. Aspects such as rationale, developing a treatment plan, practical issues interfering with treatment, psychoeducation, early symptom change are all likely linked with CEQ scores. The rationale for exposure therapy can increase patients’ credibility ratings [56]. In fact, providing patients with a sound CBT rationale could also result in increases in self-efficacy for anxiety change, confidence in conducting exposures, perceived helpfulness of exposure, and increased frequency of exposure [57].

It could also be that the predictive effect of CEQ is mediated by other factors, for instance patient adherence [58]. In this trial there was a significant correlation of 0.35 between the two constructs, which is consistent with previous research [35]. However, LeBeau et al. [34] found a poor correlation between expectancy and homework compliance, and they suggested that the two could represent unique constructs of treatment engagement, with compliance being more important. The timing of CEQ assessment is also likely an important factor in such research and CEQ scores are subject to change during the first sessions. Research has found that pre-treatment expectancy for anxiety change was not associated with OCD treatment outcome but increases in such expectancies during the first four session were [59]. This is in line with related research [40]. Expectancy for anxiety change was negatively associated with severity of OCD, depression, and anxiety [59]. However, establishing consistent predictors of credibility/expectancy has proven difficult, but another study also found a significant association with severity of depression [60].

This study has several limitations. The sample does not represent patients with OCD who have previously received other forms of treatment (e.g., SSRIs). Although the sample was categorized as difficult-to-treat, it is important to note that they were not treatment resistant (as defined by non-response to two evidence-based treatments for OCD), and that the criterion of at least 6 h of ERP does not match most ERP manuals. Despite instructing patients to focus exclusively on the psychological intervention when completing the CEQ, the presentation of both psychological and pharmacological components may have influenced their treatment expectations and perceptions. This dual presentation could have introduced confounding variables, potentially affecting the validity of our findings regarding the predictive value of credibility and expectancy. Additionally, all participants were Norwegian adults, and the study’s findings may not be generalizable to other cohorts of patients with OCD, emphasizing the need for further investigation in various populations. An important limitation is that the study did not include an active control group. Consequently, whether the results are unique to the B4DT program or if they are applicable to other types of treatments remains unknown.

## Conclusions

Patients displayed high levels of treatment credibility and strong expectancy, despite previous treatment challenges. Notably, the CEQ score was consistently associated with OCD symptom improvement at posttreatment, at the 3-month follow-up, and at the 1-year follow-up, even after controlling for other important factors. Furthermore, CEQ scores were also associated with enhanced work and social functioning at the 1-year follow-up. In summary, the findings suggest that treatment credibility and expectancy could be important to address before exposure treatment is started.

## Data Availability

The data that support the findings of this study are not openly available due to reasons of sensitivity and are available from the corresponding author upon reasonable request. Data are located in controlled access data storage at Haukeland University Hospital.

## Abbreviations

B4DT: Bergen 4-day treatment
CBI-I: Clinical Global Impression– Improvement
CBT: Cognitive Behavioral Therapy
CEQ: Credibility/Expectancy Questionnaire
cET: Concentrated Exposure Therapy
CONSORT: Consolidated Standards of Reporting Trials
DSM-5: Diagnostic and Statistical Manual of Mental Disorders-Fifth Edition
EM: Expectation Maximization
ERP: Exposure Therapy with Ritual Prevention
GAD-7: Generalized Anxiety Disorder 7-item scale
MCAR: Missing Completely at Random
OCD: Obsessive-Compulsive Disorder
PHQ-9: Patient Health Questionnaire-9
REK: Regional Committee for Medical Research Ethics
SSRI: Selective Serotonin Reuptake Inhibitor
WSAS: Work and Social Adjustment Scale
Y-BOCS: Yale-Brown Obsessive Compulsive Scale
